# Complete Genome Sequence of Vibrio anguillarum Nontailed Bacteriophage NO16

**DOI:** 10.1128/MRA.00020-19

**Published:** 2019-04-11

**Authors:** Panos G. Kalatzis, Alexander B. Carstens, Pantelis Katharios, Daniel Castillo, Lars H. Hansen, Mathias Middelboe

**Affiliations:** a Marine Biological Section, University of Copenhagen, Helsingør, Denmark; b Department of Environmental Science, Aarhus University, Roskilde, Denmark; c Institute of Marine Biology, Biotechnology, and Aquaculture, Hellenic Centre for Marine Research, Heraklion, Crete, Greece; Loyola University Chicago

## Abstract

A rare nontailed virus designated NO16 was isolated against Vibrio anguillarum, a major aquaculture pathogen for both fish and shellfish. Here, we announce the 10,594-bp genome sequence of *Vibrio* phage NO16 with a 23-gene content.

## ANNOUNCEMENT

*Vibrio anguillarum* is a pathogenic bacterium of both cultured fish and shellfish (1, 2). The host of bacteriophage NO16 is V. anguillarum strain A023 (GenBank accession numbers CP010036 and CP010037), isolated from turbot in Spain (3).

Bacterial cells were cultured in liquid medium containing 0.5% tryptone (Difco), 0.1% yeast extract (Difco), and 2% sea salts (Sigma-Aldrich), and PFU were picked from the bacterial lawn produced by the double-agar-layer method (4). Phage DNA was extracted following the protocol listed in reference 5 and was sequenced from two single plaques using the MiSeq platform (Illumina, San Diego, CA, USA) as 2 × 250-bp paired-end reads according to the direct plaque sequencing (DPS) protocol (5) using 0.1% SDS instead of 1%. *De novo* assembly of the 548,174 reads (coverage, 1,014.74×) was performed using Genomic Workbench 9.5.3 (CLC Bio, Aarhus, Denmark). Briefly, reads were trimmed using the trim sequences tool (default settings), and overlapping reads were merged using the overlapping pairs tool (mismatch cost, 2; minimum score, 8; gap cost, 3; maximum unaligned end mismatches, 0) (6). The bacterial host’s reads were removed using the map to reference tool. Circularity of the genome was confirmed by two independent approaches, (i) restriction enzyme digestion of the genome using different digestion sites followed by analysis of the size of the resulting segments and (ii) PCR amplification of specific sequences in the genome representing the site circularization (i.e., a sequence that would be amplified only if the genome was circular). Restriction enzyme ClaI (restriction site ATCGAT) produced two DNA bands on an agarose gel, whereas PCR with specific primers (forward, TGCCGGACAGAATCGAACTC; reverse, ATGCGGAGGACACGACATGA) amplified, as expected, a 625-bp DNA fragment between the phage’s genomic ends. Hence, bacteriophage NO16 has a 10,594-bp circular double-stranded DNA (dsDNA) genome with a GC content of 47.4%.

The 23 genes of the bacteriophage were predicted by Glimmer 3 (7), and they were then annotated with Rapid Annotations using Subsystems Technology (RAST) (8) and protein fold recognition server Phyre2 (9). DNA-binding protein (gene 6), *S*-adenosylhomocysteine hydrolase (gene 7), phage protein (gene 11), double jelly roll (DJR) capsid protein (gene 19), and ATPase (gene 21) are the 5 genes with some attributed function, whereas the remaining 18 open reading frames (ORFs) are hypothetical proteins. The presence of gene 19 classifies NO16 in the lineage of DJR viruses, of which very few marine members have been characterized so far (10, 11). In NCBI, the genome of NO16 was found in chromosome II of V. anguillarum strains 87-9-116, NB10, and VIB18 (query coverages of 98%, 98%, and 52%, percent identities of 99.65%, 99,65%, and 99.91%, and GenBank accession numbers CP010045, LK021129, and CP011437, respectively), suggesting that it follows a temperate life cycle, although no integrase gene has been identified in its genome. With the signature gene of the DJR viral lineage (gene 19) as a query, the most closely related phage genomes are those of PM2 (12–14) and Cr39582 (11) (query coverages of 94% and 94%; percent identities of 35.80% and 35.16%, and GenBank accession numbers NC_000867 and MG966533, respectively). Additionally, the DJR genes of several *Vibrio* phages recently published by Kauffman and colleagues (10) have similarities of 33% and below at query coverages of 96 to 97%.

### Data availability.

The genome sequence of bacteriophage NO16 was submitted to GenBank under the accession number MH730557, whereas the raw reads were uploaded to the European Nucleotide Archive (ENA) under the accession number PRJEB30917.

## References

[1] Rønneseth A, Castillo D, D'Alvise P, Tønnesen Ø, Haugland G, Grotkjaer T, Engell-Sørensen K, Nørremark L, Bergh Ø, Wergeland HI, Gram L 2017 Comparative assessment of *Vibrio* virulence in marine fish larvae. J Fish Dis 40:1373–1385. doi:10.1111/jfd.12612. 28160295

[2] Frans I, Michiels CW, Bossier P, Willems KA, Lievens B, Rediers H 2011 *Vibrio anguillarum* as a fish pathogen: virulence factors, diagnosis and prevention. J Fish Dis 34:643–661. doi:10.1111/j.1365-2761.2011.01279.x. 21838709

[3] Castillo D, Alvise PD, Xu R, Zhang F, Middelboe M, Gram L 2017 Comparative genome analyses of *Vibrio anguillarum* strains reveal a link with pathogenicity traits. mSystems 2:e00001-17. doi:10.1128/mSystems.00001-17. 28293680 PMC5347184

[4] Kutter E 2009 Phage host range and efficiency of plating. Methods Mol Biol 501:141–149. doi:10.1007/978-1-60327-164-6_14. 19066818

[5] Kot W, Vogensen FK, Sørensen SJ, Hansen LH 2014 DPS: a rapid method for genome sequencing of DNA-containing bacteriophages directly from a single plaque. J Virol Methods 196:152–156. doi:10.1016/j.jviromet.2013.10.040. 24239631

[6] Carstens AB, Kot W, Lametsch R, Neve H, Hansen LH 2016 Characterisation of a novel enterobacteria phage, CAjan, isolated from rat faeces. Arch Virol 161:2219–2226. doi:10.1007/s00705-016-2901-0. 27231007

[7] Delcher AL, Bratke KA, Powers EC, Salzberg SL 2007 Identifying bacterial genes and endosymbiont DNA with Glimmer. Bioinformatics 23:673–679. doi:10.1093/bioinformatics/btm009. 17237039 PMC2387122

[8] Aziz RK, Bartels D, Best AA, DeJongh M, Disz T, Edwards RA, Formsma K, Gerdes S, Glass EM, Kubal M, Meyer F, Olsen GJ, Olson R, Osterman AL, Overbeek RA, McNeil LK, Paarmann D, Paczian T, Parrello B, Pusch GD, Reich C, Stevens R, Vassieva O, Vonstein V, Wilke A, Zagnitko O 2008 The RAST server: Rapid Annotations using Subsystems Technology. BMC Genomics 9:75. doi:10.1186/1471-2164-9-75. 18261238 PMC2265698

[9] Kelley LA, Mezulis S, Yates CM, Wass MN, Sternberg MJE 2015 The Phyre2 Web portal for protein modelling, prediction, and analysis. Nat Protoc 10:845–858. doi:10.1038/nprot.2015.053. 25950237 PMC5298202

[10] Kauffman KM, Hussain FA, Yang J, Arevalo P, Brown JM, Chang WK, Vaninsberghe D, Elsherbini J, Sharma RS, Cutler MB, Kelly L, Polz MF 2018 A major lineage of non-tailed dsDNA viruses as unrecognized killers of marine bacteria. Nature 554:118–122. doi:10.1038/nature25474. 29364876

[11] Leigh BA, Breitbart M, Oksanen HM, Bamford DH, Dishaw LJ 2018 Genome sequence of PM2-like phage Cr39582, induced from a *Pseudoalteromonas* sp. isolated from the gut of *Ciona robusta*. Genome Announc 6:e00368-18. doi:10.1128/genomeA.00368-18. 29798916 PMC5968719

[12] Espejo RT, Canelo ES 1968 Properties of bacteriophage PM2: a lipid-containing bacterial virus. Virology 34:738–747. doi:10.1016/0042-6822(68)90094-9. 5655723

[13] Männistö RH, Kivelä HM, Paulin L, Bamford DH, Bamford JKH 1999 The complete genome sequence of PM2, the first lipid-containing bacterial virus to be isolated. Virology 262:355–363. doi:10.1006/viro.1999.9837. 10502514

[14] Espejo RT, Canelo ES, Sinsheimer RL 1969 DNA of bacteriophage PM2: a closed circular double-stranded molecule. Proc Natl Acad Sci U S A 63:1164–1168. doi:10.1073/pnas.63.4.1164. 5260915 PMC223444

